# Supplementary material for: Deep Learning for Rapid Identification of Microbes Using Metabolomics Profiles
Source: Metabolites. 2021 Dec 13;11(12):863. doi: 10.3390/metabo11120863 (PMC8704490; doi:10.3390/metabo11120863)
Supplement: Supplementary file 1 [file metabolites-11-00863-s001.zip › metabolites-1515895-supplementary.pdf]

Deep Learning for Rapid Identification of Microbes Using Metabolomics Profiles

Danhui Wang, Peyton Greenwood, and Matthias S. Klein*

Department of Food Science and Technology, The Ohio State University, Columbus, OH, USA

*Address correspondence to klein.663@osu.edu

Supplemental Materials

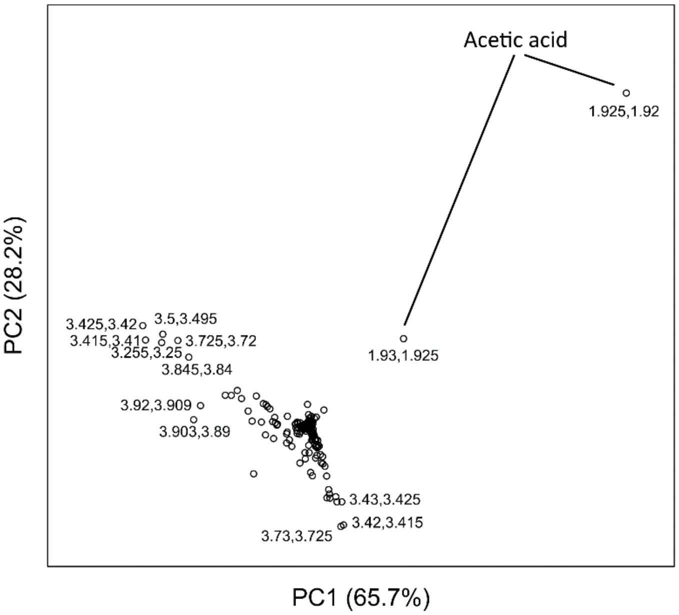

Figure S1. Loadings plot of the Principal Component Analysis (PCA). Given are left and right borders of the respective bin [ppm].

Table S1. Metabolite identities of signals significantly increasing during microbial growth.

Metabolite	Chemical shift [ppm][1]
1-propanol	0.91,0.905; 0.905,0.9; 0.895,0.89; 0.88,0.875
acetate	1.95,1.945; 1.945,1.94; 1.94,1.935; 1.935,1.93; 1.93,1.925; 1.925,1.92; 1.92,1.915; 1.915,1.91; 1.91,1.905
ethanol	1.2,1.195; 1.195,1.19; 1.19,1.185; 1.185,1.18; 1.18,1.175
formate	8.47,8.465; 8.455,8.45; 8.45,8.445
fumarate	6.53,6.525; 6.525,6.52
indole	7.73,7.725; 7.725,7.72; 7.72,7.715; 7.57,7.565; 7.565,7.56; 7.27,7.265; 7.265,7.26; 7.26,7.255; 7.17,7.165
lactate	1.35,1.31
spermidine	3.06,3.055; 1.78,1.775
succinate	2.38,2.375

[1]Given are left and right borders of the respective bin [ppm]
